# N-methyl-bacillithiol, a Novel Thiol from Anaerobic Bacteria

**DOI:** 10.1128/mBio.02634-18

**Published:** 2019-01-15

**Authors:** Gerald L. Newton, Mamta Rawat

**Affiliations:** a Division of Biological Sciences, University of California, San Diego, San Diego, California, USA; b Department of Biology, California State University—Fresno, Fresno, California, USA

**Keywords:** *Chlorobium*, N-methyl-bacillithiol, anaerobes, bacillithiol, glutathione, thiols

## Abstract

J. Hiras, S. V. Sharma, V. Raman, R. A. J. Tinson, et al. (mBio 9:e01603-18, 2018, https://doi.org/10.1128/mBio.01603-18) report on the identification of a novel thiol, N-methyl-bacillithiol (N-Me-BSH), in the green sulfur bacterium Chlorobium tepidum. In N-methyl-bacillithiol, the amine of the cysteine is methylated by a novel *S*-adenosylmethioneine transferase designated N-methyl-bacillithiol synthase A (NmbA). The Hiras et al. study is significant because it is the first report of the presence of N-Me-BSH in anaerobic bacteria.

## COMMENTARY

**FIG 1:**
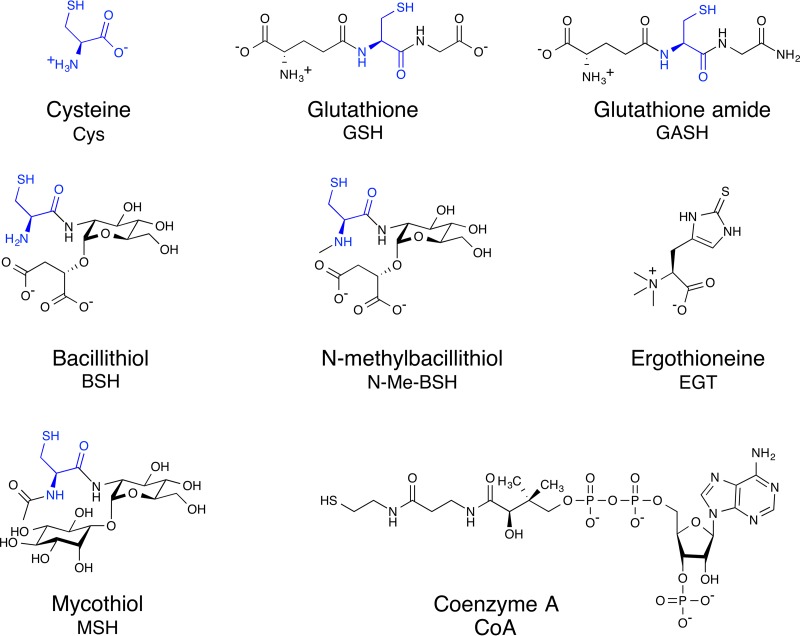
Low-molecular-weight thiols in bacteria: N-Me-BSH, Cys, GSH, GASH, BSH, MSH, EGT, and CoA. The cysteine portion of the molecule is shown in blue.

Low-molecular-weight (LMW) thiols are small molecules, often containing cysteine (Cys), which serve as a redox buffer in aerobic cells. These thiols are also an important storage form for cysteine, since cysteine autooxidizes in the presence of metal ions, reducing free ferric iron or cupric ion to the ferrous and cuprous forms. These reduced metal ions participate in the Fenton reaction redox cycling in the presence of hydrogen peroxide, increasing hydroxyl radicals within the cell (1). The best studied LMW thiol is the tripeptide GSH (γ-L-glutamyl-L-cysteinyl-glycine), whose role has been extensively detailed in plants and animals. Early assays of LMW thiols measured by total thiol titration or glutathione reductase assays demonstrated that GSH is the major LMW thiol in cyanobacteria, purple bacteria, and eukaryotes containing mitochondria or chloroplasts (2), but not in most Gram-positive bacteria or anaerobes (3). With the development of LMW thiol labeling with monobromobimane (mBBr) and HPLC analysis of these thiols (4), Fahey and colleagues were able to demonstrate that unidentified thiols other than the ubiquitous cysteine (Cys), coenzyme A (CoA), and GSH were present in bacteria (5, 6). Subsequent structural characterization of the major LMW thiol in high-GC Gram-positive actinomycetes resulted in the identification of mycothiol (MSH) (7–9) and bacillithiol (BSH) in low-GC Gram-positive *Firmicutes* and *Deinococcus* (10). These two LMW thiols differ substantially in structure from GSH in having a backbone of cysteine amide bonded to glucosamine (Fig. 1). In MSH, glucosamine is linked to inositol, while in BSH, it is linked to malate. However, one feature that these novel thiols share with GSH is that the cysteine moiety is linked by an unusual γ-glutamyl-cysteine or cysteinyl-glucosamine amide bond, which is resistant to cellular peptidases. Dedicated amide hydrolases, such as γ-glutamyl transpeptidase or MSH/BSH conjugate amidase, are required to release cysteine for metabolism from GSH, MSH, or BSH, respectively.

The prevailing paradigm suggests that LMW thiols, particularly GSH, evolved to protect against reactive oxygen species present in the oxidizing environment. However, oxygenation of the atmosphere by the cyanobacteria occurred 2.4 billion years ago. Prior to the accumulation of atmospheric oxygen, extensive diversity already existed among prokaryotes. Early earth was populated with heterotrophs, which used a wide variety of electron acceptors instead of oxygen in anaerobic respiration, and autotrophs, which included chemoautotrophs and photoautotrophs. The anaerobic photoautotrophs performed anoxygenic photosynthesis, using hydrogen sulfide/thiosulfate/sulfur in the place of water as a reductant, and the waste product was an oxidized S species instead of oxygen. Surveys of anaerobic bacteria indicate that the ubiquitous LMW thiols, Cys and CoA, are present in smaller amounts than aerobic or facultative bacteria, GSH is absent, and novel thiols have been detected in the heterotrophs Clostridium pasteurianum and Clostridium kluyveri and the photoautotrophs Chlorobium thiosulfatophilum, Chloroflexus aurantiacus, Allochromatium vinosum, Marichromatium gracile, and Isochromatium buderi (5, 6, 11). The presence of these novel LMW thiols in these anaerobic bacteria suggested that LMW thiols serve functions other than protection against oxidative stress.

In 1996, Bartsch and colleagues identified glutathione amide (GASH), as the major LMW thiol in the anaerobic purple sulfur bacterium Gammaproteobacteria, Allochromatium vinosum (formerly Chromatium vinosum) (12). This bacterium is able to grow heterotrophically and also autotrophically through anoxygenic photosynthesis (13). A second unknown thiol from A. vinosum and Marichromatium gracile cultured in H_2_S was determined to be the perthiol of GASH, GASSH. Under heterotrophic conditions, GASH was the dominant LMW thiol, but the perthiol, GASSH, was present in higher quantities under anoxygenic photosynthesis conditions. This increase in perthiol concentration suggested that its function may be to transfer sulfur from the cytoplasm to the periplasmic space where sulfur (S0) accumulates in globules in *A. vinosum.* Interestingly, GASH autoxidizes in air-saturated solutions sevenfold slower than GSH in the presence of catalytic amounts of copper ion (12). This suggests a role for the glycine carboxylate of GSH in binding metal ions and thiol autoxidation. A glutathione amide disulfide (GASSGA) reductase utilizing NADH instead of NADPH and a GASH-dependent peroxiredoxin have been isolated from M. gracile, indicating a competent pathway for hydroperoxide reduction in these anaerobic and microaerophilic bacteria (14).

Now, Hiras and colleagues report the identification and characterization of a novel thiol from the green sulfur bacterium Chlorobium tepidum (15). Like *Chromatium* sp., *Chlorobia* perform anoxygenic photosynthesis, oxidizing reduced sulfur compounds for CO_2_ fixation and are obligate anaerobes. Compared to the photosynthetic *Proteobacteria* such as *Allochromatium* sp., the green sulfur bacteria branch shallow within the phylogenetic tree of eubacteria (16). *Chlorobia* and *Chloroflexus* dominate the H_2_S-containing geothermal environments found in areas such as Mammoth hot springs in Yellowstone National Park. Hiras et al. (15) report that C. tepidum, Chlorobium phaeobacterioides, and *Prosthecochloris* sp. strain CB11 produce N-methyl-bacillithiol (N-Me-BSH) when grown photoautotrophically under anaerobic conditions. The genes responsible for the biosynthesis of BSH were identified by sequence comparison with orthologs in Bacillus subtilis, and the requirement for BSH in N-Me-BSH biosynthesis was confirmed by deletion of *bshB,* coding for the second step in BSH biosynthesis. N-methylation of the cysteine amine is rare and is catalyzed by an *S*-adenosylmethioneine (SAM)-containing enzyme (CT1040) designated N-methyl-bacillithiol synthase A (NmbA). Mutant strains with disruptions in genes involved in BSH biosynthesis or *nmbA* grew 20% slower than the wild-type strain in media containing sulfide or thiosulfate. Interestingly, the cultures grown in low light contained fivefold more N-Me-BSH than cells grown in standard or high-light conditions.

Hiras and colleagues (15) also searched complete genome sequences with the BSH biosynthesis genes *bshA*, *bshB,* and *bshC* as well as *nmbA* for bacteria that could potentially produce BSH or N-Me-BSH. Orthologs of *nmb*A along with genes involved in BSH production were found in a representative *Chlamyidae* (Waddia chondrophila), *Bacteriodetes* (*Polaribacter* sp. strain MED152), *Acidobacteria*, and the *Firmicutes*. *Polaribacter* sp. strain MED152 was confirmed to produce N-Me-BSH by monobrombimane and HPLC analysis. Since species from the *Bacteroidetes* and *Chlorobi* phyla branch very closely together in phylogenetic trees, the presence of BSH and N-Me-BSH was not surprising (16, 17). The remaining bacteria have the potential to produce N-Me-BSH but await validation. Hiras et al. (15) identified orthologs of *nmbA* in the phylum *Clostridia*; however, the *Clostridia* phylogeny has recently undergone a major reevaluation based on whole-genome sequences (18). The strains identified as *Clostridia* by Hiras et al. have been reclassified and are closer in lineage with the *Chlorobia*, and thus, the distribution of N-Me-BSH is not as wide as asserted. Nevertheless, the production of cysteinyl-glucosamine (Cys-GlcN)-based LMW thiols (BSH, N-Me-BSH, and MSH) appears to be very common among Gram-positive and anaerobic bacteria.

Recently, Seebeck and colleagues (19) reported on the presence of ergothioneine (EGT), a thiolimidizole derivative of histidine, using mass spectrometry in Chlorobium limicola. They demonstrated that the key reactions in EGT biosynthesis, the trimethylation of histidine to form hercynine and the sulfoxidation of hercynine to form hercynylcysteine sulfoxide anaerobically, are catalyzed by the product of Clim_1148 and Clim_1149, respectively. Orthologs of Clim_1148 and Clim_1149 are not present in the *C. tepidum* genome, and Hiras et al. (15) did not detect EGT in *C. tepidum.* Previous surveys based upon mBBr labeling also often failed to detect EGT, since EGT quenches the fluorescence of the bimane derivative and needs to be present at high levels for detection.

### Anaerobic functions of LMW thiols: more than protection from oxidative stress.

What is the role of N-Me-BSH in *Chlorobi* and other anaerobes? Hiras et al. (15) report that N-Me-BSH levels are affected by light conditions and growth phase. It likely does not primarily protect against oxidative stress in an organism growing in H_2_S-saturated (anaerobic) water. Its role as an S(0) shuttle is discounted by Hiras et al., since N-Me-BSH-containing species such as *Polaribacte*r do not have sulfur-based metabolism, and there is no accumulation of S(0). The perthiol of N-Me-BSH may be involved in sulfur transfer as in *A. vinosum* (12), and quantification of this perthiol and/or discovery of N-Me-BSH disulfide reductase may shed further light on its role in sulfur metabolism.

An important role for LMW thiols that predates the protection against oxidative stress is the detoxification of electrophiles, including epoxides, enones, and other sulfhydryl reactive agents. LMW thiols are nucleophiles able to react with electrophiles spontaneously or through catalysis by *S*-transferases, forming *S*-conjugates. Bacillithiol *S*-transferases, which form C-S bonds with electrophiles, have been identified in Bacillus subtilis (20, 21) and Staphylococcus aureus (22–24). Using the DinB model protein Yfit (Bacillus subtilis BstA) to query the Superfamily database (http://supfam.org/SUPERFAMILY/). one homolog (e.g., CT1840) is found in each of the represented *Chlorobium* genomes; Chlorobium tepidum TLS, C. limicola DSM245, and C. phaeobacteriodes DSM261. Detoxification in *Chlorobi* may be important since each of the above strains produces BSH or N-Me-BSH as a cofactor and has a DinB bacillithiol *S*-transferase homolog within the small 2.15-Mbp genome. The *S*-conjugates are either excreted or processed to mercapturic acids by bacillithiol conjugate amidases, which are then excreted in S. aureus (25). Homologs of Mycobacterium tuberculosis MSH conjugate amidases are found in antibiotic biosynthesis gene clusters, and mercapturic acid derivatives of ansamycin and naphthoquinone have been found in fermentation broths, indicating that the structurally similar MSH is involved in detoxification of thiol-reactive antibiotics (26).

Endogenous electrophiles, such as methylglyoxal, are also detoxified in an LMW thiol-dependent manner (27), and orthologs of other genes that code for proteins are involved in detoxification of endogenous electrophiles, such as thiol-dependent formaldehyde dehydrogenases, are present in BSH-containing organisms (28). Presumably, these enzymes will be found in N-Me-BSH-containing organisms. Recently, another important function has been revealed for the LMW thiols in the biosynthesis of secondary metabolites. The LMW thiols, ergothioneine and MSH, can serve as cofactors in the formation of C-S bonds in the biosynthesis of the antibiotic lincomycin (29). N-Me-BSH-dependent detoxification and secondary metabolite production may play an important role in *Chlorobi* and other anaerobes.

Another function for N-Me-BSH may be in metal homeostasis. BSH-based metal chelation was predicted based on its structure (10) and then demonstrated experimentally with affinity measurements for Zn (β_2_=1.9 × 10^12^ M^−2^) (30) and Cu (β_2_= 4.1 ± 1.5 × 10^17^ M^−2^) (31). By comparison, GSH is a much weaker chelator of Zn with a binding affinity of ∼3 × 10^4^ M^−1^ (30). BSH is able to compete with metallochaperones for Zn (30) and competes with the metalloregulator CopA (32) and Cu exporter CopZ for Cu in Bacillus subtilis (31). The metal speciation of BSH with Cu ions and CopA/CopZ were detailed with mass spectrometry methods showing previously unknown complexes in elegant studies by Le Brun and colleagues (31, 32). The affinity of BSH for Zn or Cu ions indicates that free metal ions in bacteria producing BSH will be in the nanomolar-to-picomolar range. How the presence of the methyl group in N-Me-BSH alters the affinity for metals is an interesting question.

Despite the absence of a clear function for N-Me-BSH, the study by Hiras et al. (15) adds to the panoply of LMW thiols in bacteria. Further investigation of physiochemical properties of this thiol may provide insight into its exact role in aerobic and anaerobic bacteria.
